# Morphine-Induced Fever: A Case Series

**DOI:** 10.7759/cureus.24402

**Published:** 2022-04-22

**Authors:** Manisha Bhagat, Saurabh Suman, Kartik C Besra, Tushar Kumar, Shio Priye, Pradip K Bhattacharya, Ladhu Lakra

**Affiliations:** 1 Anesthesiology, Rajendra Institute of Medical Sciences, Ranchi, IND; 2 Critical Care, Rajendra Institute of Medical Sciences, Ranchi, IND

**Keywords:** post operative fever, cervical cord, intervertebral disc, prostheses and implants, venous thromboembolism (vte), cox-2 inhibitors, postopertive infection, non-steroidal antiinflammatory drugs, opioid use

## Abstract

The most common cause of postoperative fever is infection. Other causes include cancer, iatrogenic causes, venous thromboembolism, secondary to prosthetic implants, and pyrexia of unknown origin. Here, we describe five cases of opioid-induced pyrexia. In all cases, an injection of morphine was given for postoperative analgesia and all those patients developed fever. All the possible causes of fever were excluded and then opioid was substituted with non-steroidal anti-inflammatory agents. Fever subsided in all the cases. Cessation of the offending drug led to the resolution of the fever in all five cases, and the patient required subsequent supportive care. However, adjunctive pharmacotherapy may also be needed in some patients.

## Introduction

Fever has myriad causes, and medications are one of them. They are never the obvious etiological agents. Drug administration can cause fever by disrupting homeostasis, interfering with peripheral heat dissipation, increasing metabolism rate, inducing a cellular or humoral immune response, mimicking an endogenous pyrogen, or causing tissue damage [1]. Drug-induced fever is most commonly the consequence of a hypersensitivity reaction, and its features resemble those of an allergic reaction. Opioids are the most commonly used drugs for postoperative acute pain management. Opioid-induced fever, especially with morphine, is an extremely rare and poorly known symptom. Here, we describe five cases wherein patients who underwent surgery between January 2020 and December 2021 and were administered morphine for postoperative analgesia developed fevers during the postoperative period. During the two-year period, different batches of injection morphine from different manufacturers were used. We conducted more than 20,000 major cases in two years and identified five patients suspected of developing a morphine-induced fever. 

## Case presentation

The patient or guardian provided written informed consent for publication. The case presentation is as per CARE guidelines.

Case 1 

A previously healthy three-year-old female child was admitted to our hospital for a huge Wilms tumor of the left kidney that presented as a swelling in the left lumbar region about one year ago. On examination, she was pale and cachectic and weighed only 12 kg. Surgery was planned after several cycles of chemotherapy had reduced the abdominal swelling to some extent. A pre-anesthetic evaluation was performed, and the patient was prepared for surgery. The procedure was performed under general anesthesia as per plan, and the patient was transferred to the postoperative ICU, where she was on elective mechanical ventilation. She was sedated with midazolam infusion at the rate of 0.15 mg/kg/hr and intravenous (IV) morphine was administered at the rate of 0.5 mg per hour. While the first 12 hours were uneventful, she gradually developed a fever. That started at 100 °F and sored up to 105 °F in the next 8 hours. Her total leukocyte count was 6,500/cu mm and C-reactive protein was 4 mg/dL. As all other causes of fever were evaluated such as infection, tumor handling, IV fluid-induced, antibiotics, and stress. Paracetamol 10 mg/kg IV helped in reducing fever but normothermia could not be achieved. Injection morphine was replaced by paracetamol 20 mg/kg IV for analgesia. Her fever subsided in the next six hours and did not happen again. She was extubated 24 hours after arriving at the ICU and was later discharged on day 10 with follow-up advice.

Case 2

A 46-year-old female with a pancreatic tumor underwent a Whipple procedure under general anesthesia. The surgery was uneventful, the patient was transferred to the postoperative ICU, and an epidural catheter was placed to alleviate postoperative pain. Morphine, 10 mg diluted in 10 mL of normal saline, was given slowly via the epidural catheter over 12 hours. The next day, she developed a fever of 102 °F, which gradually increased to 104 °F the same day. Causes of fever were evaluated and it was found that there was no history of CBD stent placement. Her blood investigations showed hemoglobin of 9.4 mg/dL and TLC of 8,400/cu mm. Both blood and urine cultures and a fever workup were unremarkable. Morphine for analgesia was replaced by bupivacaine 0.08% at the rate of 5 mL per hour. Her pain remained well-controlled, and the fever subsided in the next eight hours.

Case 3

A 27-year-old male with cervical cord compression at the level of C5-C6 underwent cord decompression under general anesthesia. After surgery, the patient was transferred to the neurosurgical ICU with an ET tube in situ and was administered morphine and midazolam for sedation and analgesia. After 6-8 hours, the patient developed a fever of up to 104.2 °F and a complete blood count revealed total WBC to be 9,700/cu mm and C-reactive protein was 10 mg/dL. His fever was controlled with IV paracetamol 1 g every eight hours, and it subsided in the next four hours with no recurrence. The patient was extubated the next day, and infusion of midazolam and morphine were replaced by 50 mg of tramadol injections every six hours. In this case, we did not stop morphine infusion as it was not anticipated as a causative agent. Fever might be due to surgical stress or drug induced but it remained unclear.

Case 4

A 45-year-old male with an L4-L5 prolapsed intervertebral disc underwent decompression and fusion under general anesthesia. He was extubated and transferred to the postoperative ICU. Morphine, 4 mg every four hours, was given intraoperatively and in the postoperative period for analgesia. The patient developed a fever of 104.6 °F about six hours after surgery, which subsided with IV paracetamol 1 g. However, fever recurred every six hours, and the total leukocyte count was 8,400/cu mm and C-reactive protein after 24 hours was 15 mg/dL. At 48 hours after surgery, morphine was stopped because opioid-induced fever was suspected, and this resolved the fever.

Case 5

A 55-year-old female with cervical cord compression at the level of C3-C5 underwent cord decompression and fixation under general anesthesia. After surgery, the patient was transferred to the neurosurgical ICU with an ET tube in situ and was administered morphine and midazolam for sedation and analgesia. After six hours, the patient developed a fever of up to 103.4 °F and a complete blood count revealed total WBC to be 10,230/cu mm and C-reactive protein at 24 hours was 10 mg/dL. Her fever was controlled with IV paracetamol 1 g every eight hours, and it subsided in the next four hours. The patient was extubated the next day and infusion of midazolam and morphine were replaced by 50 mg of tramadol injections every six hours. There were no fever after cessation of morphine.

The demographic profile and relevant findings are shown in Table 1.

**Table 1:** Patient profile and relevant findings.

Sno	Age (years)	Sex	Diagnosis	Procedure	Offending drug	Route of administration	Total leukocyte count ( per cubic mm)	Maximum temperature (°F)
1	3	F	Wilms tumor	Exploratory laprotomy with tumor excision	Morphine	Intravenous	6500	105
2	46	F	Pancreatic tumor	Whipple procedure	Morphine	Epidural	8400	104
3	27	M	C5-C6 cervical cord compression	Anterior corpectomy	Morphine	Intravenous	9700	104.2
4	45	M	L4-L5 prolapsed intervertebral disc	Decompression and fusion	Morphine	Intravenous	8400	104.6
5	55	F	C3-C5 cervical cord compression	Decompression and fixation	Morphine	Intravenous	10230	103.4

## Discussion

Morphine-induced pyrexia is a rare and poorly understood symptom that is predominantly a diagnosis of exclusion. Body temperature is controlled in a narrow range to enable normal physiological processes. The hypothalamus has a very crucial role to play in maintaining basic homeostatic functions as it processes all the information on body heat and integrates them to provide an autonomous, endocrine, motoric, and behavioral response to the changes in the environment [2]. According to Romanowsky et al., there are independent thermoeffector loops with afferent and efferent impulsion pathways [3]. Depending on conditions, body temperature either increases or decreases, affecting the balance point of thermoeffector networks (previously setpoint) that work as a “thermostat” to determine central body temperature beyond physiological values, i.e., between 36.1°C and 37.4°C.

Fever in the postoperative period has numerous causes including changes in the abilities of immune response (cancer itself, malnutrition, implemented therapy), mass effects of cancer progression (tissue proliferation, fistulas, and effect on natural biological barriers), implantation of foreign objects, and their effect on the mucosa, repeated antibiotic use, and cessation of prescribed drug regimens (opioids, benzodiazepines) [4]. Pyrogens can directly affect the thermoregulatory center in the hypothalamus and, especially, on the front preoptic nuclei, thus enhancing the thermal value by altering the activity of the brain cyclooxygenases (COX). In endothelial cells and cells adjacent to the vessel walls of the blood-brain barrier, proinflammatory cytokines instigate the production of prostaglandin E2, which is the cardinal and central mediator of pyrexia. Crucially, the synthesis of prostaglandin E2 is catalyzed by, among others, phospholipase A2 and COX. In patients with brain metastases, paraneoplastic fever can also occur from direct damage to the neural tissue and resulting in the activation of phospholipase A2 [5].

It is hard to distinguish fevers of infectious origin from those of other causes, such as drug-induced or paraneoplastic fever. Paraneoplastic fever is an important cause of fever in cancer patients where the presence of an infection is not always necessary. Molecular factors that cause fever in cancer patients are interleukin 1α (IL-1α), interferon α (INF-α), tumor necrosis factor α (TNF-α), IL-6, oncostatin-M, corticotrophin, and leukemia inhibitory factor [6]. Typically, fever due to infections manifests as temperature surges that are accompanied by shivering and sweating; in contrast, paraneoplastic fever is characterized by a constant sense of heat and sweating, generally without shivering.

Further, paraneoplastic fever does not respond to acetylsalicylic acid or acetaminophen, unlike fever due to infections. Paraneoplastic fever reduces after administration of naproxen (naproxen test) or other non-steroidal anti-inflammatory drugs (NSAIDs), such as ibuprofen and diclofenac [6,7]. Chang’s paraneoplastic fever diagnostic criteria and Hunter's serotonin toxicity criteria need to be considered [8,9]. The criteria for paraneoplastic fever are shown in Table 2 [10].

**Table 2:** Criteria for paraneoplastic fever.

Criteria	
I	Temperature over 37.8°C at least once each day
II	Duration of fever over two weeks
III	Lack of evidence of infection on:
	A. Physical examination
	B. Laboratory examinations, e.g. sputum smears or cultures, cultures of blood, urine, stool, bone marrow, spinal fluid, and discharge from local lesions
	C. Imaging studies, e.g. chest radiograph or CT scans of the head, abdomen, and pelvis
IV	Absence of allergic mechanisms, e.g. drug allergy, transfusion reaction, or radiation and chemotherapeutic drug reaction
V	Lack of response of fever to an empiric, adequate antibiotic therapy for at least seven days
VI	Prompt, complete lysis of fever by the naproxen test with sustained normal temperature while receiving naproxen

**Figure 1:**
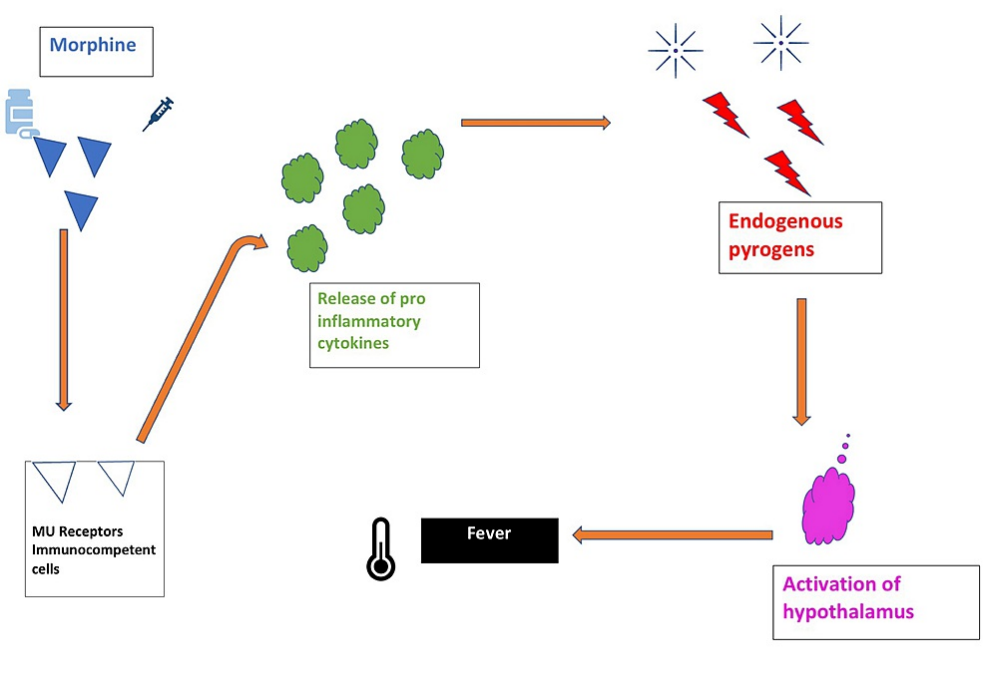
Opioid-induced fever pathway.

Opioid-induced fever is an extremely rare phenomenon and is primarily a diagnosis of exclusion. Nevertheless, analyzing the time relationship between temperature trends and opioid use can indicate opioid-related fever. The proposed mechanism underlying opioid-related fever involves stimulating opioid receptors of the mu (μ) type that are present on immunocompetent cells in some genetically susceptible individuals. This leads to the expression of proinflammatory cytokines that enhance the production of endogenous pyrogens, which then triggers the hypothalamus and cause fever (Figure 1). Opioids may also alter the spectrum of functions in the brain that is responsible for thermoregulation.

The mechanism of opioid-induced hyperthermia can be compared to that of opioid-induced hyperalgesia (OIH), which is observed in certain patients treated with opioids, and is presented as increased sensitivity to painful stimulus despite higher doses of the drug [11]. One theory suggests that, in OIH, there is an emanation in the reticular formation of “on” type neurons in lieu of “off” type neurons [1]. It is also possible that, opioids directly influence the centers responsible for thermoregulation in brain due to the obvious presence of opioid receptors in neural structures. An incomplete clarification for this is that a pure agonists of mu (μ) type receptors, i.e., those without a ceiling effect, can induce hyperthermia. Opioids, like codeine which, have a ceiling effect, but do not demonstrate this ability [12]. Neurotransmitters and neuromodulators, for example serotonin, dopamine, norepinephrine, prostaglandin E1, acetylcholine, and GABA interact with opioids and change central body temperature. Thus, as hyperthermia and hypothermia caused by an opioid are due to the action on the opioid receptor by μ, κ or delta receptor agonists, both these events can be blocked by antagonists. In both scenarios, tolerance and cross-tolerance may also develop.

## Conclusions

Morphine-induced fever is a rare clinical entity seen in hospitalized patients and currently remains a diagnosis of exclusion. We encountered only five cases over two years and with more than 20,000 cases. Intensivists should be aware of this phenomenon as a probable cause of fever in post-surgical patients, apart from infection or paraneoplastic syndromes. Differential diagnosis and clinical assessment should be the mainstay to detect opioid-induced fever. Supportive care and stopping the offending drug are advised. In some severe cases, other pharmacological support may be required. The alternative analgesics can be non-steroidal anti-inflammatory drugs in these types of post-operative patients.
